# Service evaluation to establish the sensitivity, specificity and additional value of broad-range 16S rDNA PCR for the diagnosis of infective endocarditis from resected endocardial material in patients from eight UK and Ireland hospitals

**DOI:** 10.1007/s10096-014-2145-4

**Published:** 2014-06-15

**Authors:** K. A. Harris, T. Yam, S. Jalili, O. M. Williams, K. Alshafi, T. Gouliouris, P. Munthali, U. NiRiain, J. C. Hartley

**Affiliations:** 1 Department of Microbiology, Virology and Infection Control, Great Ormond Street Hospital (GOSH) for Children NHS Foundation Trust, Great Ormond Street, London, WC1N 3JH UK; 2 Department of Infection, Southampton General Hospital, Southampton, UK; 3 Papworth Hospital NHS Trust, Cambridge, UK; 4 Bristol Public Health Laboratory, Public Health England, Bristol, UK; 5 Microbiology Department, Royal Brompton and Harefield NHS Foundation Trust, London, UK; 6 Cambridge Public Health England Microbiology and Public Health Laboratory, Addenbrooke’s Hospital, Cambridge, UK; 7 Microbiology Department, University Hospitals Coventry and Warwickshire (UHCW) NHS Trust, Coventry, UK; 8 Division of Clinical Microbiology, Galway University Hospitals, Galway, Ireland

## Abstract

Infective endocarditis (IE) can be diagnosed in the clinical microbiology laboratory by culturing explanted heart valve material. We present a service evaluation that examines the sensitivity and specificity of a broad-range 16S rDNA polymerase chain reaction (PCR) assay for the detection of the causative microbe in culture-proven and culture-negative cases of IE. A clinical case-note review was performed for 151 patients, from eight UK and Ireland hospitals, whose endocardial specimens were referred to the Microbiology Laboratory at Great Ormond Street Hospital (GOSH) for broad-range 16S rDNA PCR over a 12-year period. PCR detects the causative microbe in 35/47 cases of culture-proven IE and provides an aetiological agent in 43/69 cases of culture-negative IE. The sensitivity, specificity, positive predictive value (PPV) and negative predictive value (NPV) of the 16S rDNA PCR assay were calculated for this series of selected samples using the clinical diagnosis of IE as the reference standard. The values obtained are as follows: sensitivity = 67 %, specificity = 91 %, PPV = 96 % and NPV = 46 %. A wide range of organisms are detected by PCR, with *Streptococcus* spp. detected most frequently and a relatively large number of cases of *Bartonella* spp. and *Tropheryma whipplei* IE. PCR testing of explanted heart valves is recommended in addition to culture techniques to increase diagnostic yield. The data describing the aetiological agents in a large UK and Ireland series of culture-negative IE will allow future development of the diagnostic algorithm to include real-time PCR assays targeted at specific organisms.

## Introduction

Infective endocarditis (IE) is a rare but severe disease in Europe, and diagnosis is established using the modified Duke criteria [1–3]. Where surgically excised endocardial material is available, microbiological infection of the endocardium can be demonstrated using histopathological methods [4] and microbiological culture [1]. In cases where endocardial material is not available, blood culture is the ‘gold standard’ test for the diagnosis of IE and is used alongside echocardiographic findings, serology and other clinical features consistent with IE [1].

In recent times, broad-range bacterial polymerase chain reaction (PCR) assays have been applied to resected heart valve material, and most of the published studies show that this technique can detect the causative organism of IE in a greater number of cases than the traditional histopathological and culture-based methods [2, 5–8]. Establishing the aetiology of IE is critical for the optimal management of patients. Culture-based methods have been shown to lack both sensitivity and specificity, either due to previous antibiotic therapy or because of fastidious and difficult to culture pathogens, such as *Bartonella* species, *Tropheryma whipplei*, *Coxiella burnetii*, *Legionella* species, *Mycoplasma* species and the HACEK group of organisms [1, 2, 9]. PCR-based testing of valve material is particularly important for establishing the aetiology of culture-negative cases of IE, but may also be positive in cases of previously treated IE [10].

For more than 10 years, broad-range 16S rDNA PCR has been performed as part of the routine investigation of endocardial specimens in the Clinical Microbiology Laboratory at Great Ormond Street Hospital (GOSH). We present here the results of a clinical case-note review of 151 patients, from eight UK and Ireland hospitals, whose endocardial specimens were referred to the Microbiology Laboratory at GOSH for broad-range 16S rDNA PCR over a 12-year period. This service evaluation aimed to determine the sensitivity and specificity of broad-range 16S rDNA for diagnosing IE and to establish the additional diagnostic value of performing 16S rDNA PCR on culture-negative heart valve material in IE cases.

## Methods

### Patients

From January 2000 until December 2011, a total of 169 endocardial specimens from 151 patients from eight UK and Ireland hospitals were referred to the Microbiology Laboratory at GOSH for broad-range 16S rDNA PCR. This assay is performed as part of the routine microbiological investigation of selected endocardial specimens and the PCR results were used in patient management. One hundred and sixteen patients had a final diagnosis of IE. The proportion of male patients was 68 % and the mean patient age was 51 years (range 1–84). The proportion of patients with native valve IE was 64 %. Samples were from a highly selected group of patients chosen through local diagnostic pathways and was not a representative sample of all endocardial material sent to the laboratories. Neither antimicrobial therapy, type or duration, or timing of blood cultures before endocardial tissue was obtained was recorded.

### Clinical and microbiological data

For this service evaluation, the following clinical and microbiological data were collected from the patients’ notes:

1. Was an organism grown from either the valve or from blood cultures?
2. Did any other laboratory tests indicate the presence of infection (e.g. serological and histopathological analyses)?
3. Was the clinical diagnosis IE?

### Cultures

Samples were submitted to culture in local laboratories according to local standards in Clinical Pathology Accreditation (CPA)-accredited laboratories as part of the routine microbiological investigations. The definition of a positive culture for this study is either culture-positive endocardial tissue or blood culture-positive with a clinically determined, significant organism.

### DNA extraction and broad-range PCR

DNA extraction and broad-range 16S rDNA PCR was performed as previously described [11], with the following modifications. An additional bead-beating step was performed during DNA extraction and the PCR reaction was carried out using MolTaq 16S DNA polymerase and buffers (VH Bio, Gateshead, UK).

### Amplicon sequencing

Sequencing reactions and analysis were performed as previously described [11]. Mixed sequences were resolved by cloning the amplicon using the TOPO TA Cloning Kit (Life Technologies).

### Study design

The study was registered as a Clinical Audit at GOSH on 4th July 2012 (audit number 1105) and did not require ethical approval. The referring laboratories also registered the study as a clinical audit or case note-review in accordance with local policies. This service evaluation was designed to investigate the following:

1. Sensitivity of 16S rDNA PCR for detecting an organism in cases of culture-proven IE.
2. Additional diagnostic value of 16S rDNA PCR in cases of culture-negative endocarditis.
3. PPV, NPV, sensitivity and specificity of the PCR, using clinical diagnosis of IE as the reference standard.
4. Description of the aetiology of IE from a large UK series of culture-negative cases.

## Results

### Patients with no IE diagnosis

Thirty-five patients were not diagnosed with IE, of which 32 were PCR-negative, valve culture-negative and blood culture-negative. The remaining three patients were PCR-positive (one was also culture-positive). The organisms detected were *Micrococcus luteus* and *Propionibacterium acnes* (two patients).

### Sensitivity of 16S rDNA PCR for detecting organisms in culture-positive IE

**Fig. 1:**
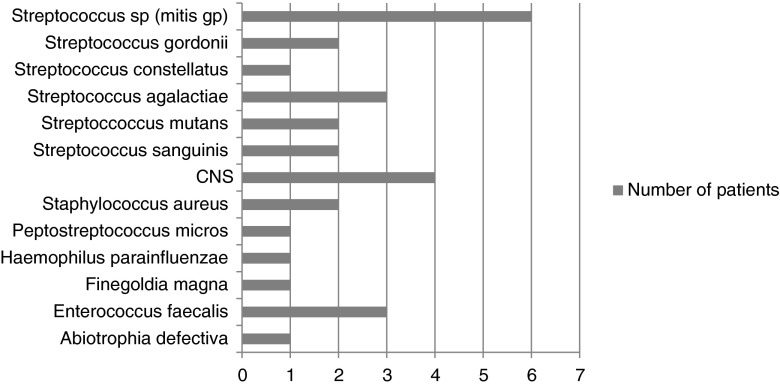
Organisms detected in 29 culture-proven infective endocarditis (IE) patients with concordant culture and polymerase chain reaction (PCR) results. CNS = coagulase-negative staphylococci

**Fig. 2:**
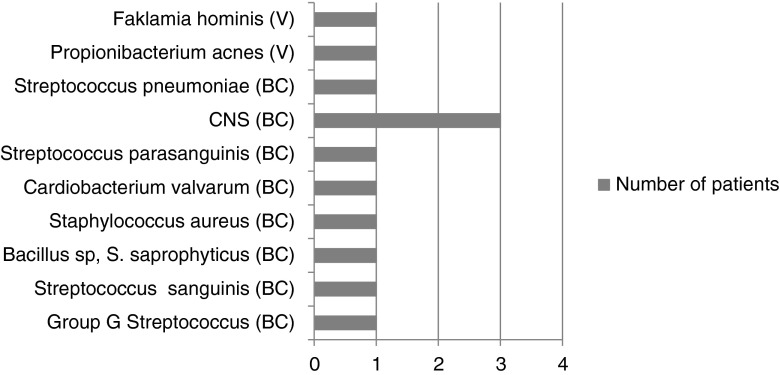
Organisms detected in 12 culture-proven IE patients with negative PCR results. *BC* blood culture, *V* valve culture, *CNS* coagulase-negative staphylococci

Forty-seven patients with a final diagnosis of IE were either blood culture- or valve culture-positive (culture-proven IE). Thirty-five of these patients were also 16S rDNA PCR-positive. PCR and culture identified the same organism in 29 of these patients (concordant results, organisms listed in Fig. 1) and different organisms in six (discordant results, organisms listed in Table 1). Twelve patients with positive cultures were 16S rDNA PCR-negative (Table 2); organisms detected by culture only are shown in Fig. 2. The sensitivity of 16S rDNA PCR in culture-proven IE is up to 75 % in this series.

**Table 1:** Organisms identified in six patients with positive but discordant PCR (V) and culture (BC or V) results

Patient	16S rDNA PCR	Culture
115	*Kytococcus schroeteri*	*Micrococcus* spp. (BC)
147	*Streptococcus sinensis*	*Streptococcus anginosus* (BC)
71	*Streptococcus bovis*	*Streptococcus oralis*, CNS (V)
74	*Streptococcus gordonii*	*Streptococcus sanguinis* (BC)
76	*Streptococcus mitis* gp + *Lactobacillus lactis*	Diphtheroids (V)
		*Pseudomonas aeruginosa* (BC)
79	*Streptococcus salivarius*	*Streptococcus vestibularis* (BC)

*BC* blood culture, *V* valve culture, *CNS* coagulase-negative staphylococci

**Table 2:** Culture and polymerase chain reaction (PCR) results from 47 patients with culture-proven infective endocarditis (IE)

	Culture (V or BC) + (concordant)	Culture (V or BC) + (discordant)	Total
16S rDNA PCR +	29	6	35
16S rDNA PCR −	0	12	12
Total	29	18	47

### Sensitivity and specificity of 16S rDNA PCR in patients with clinical diagnosis of IE

Table 3 shows the 16S rDNA PCR results in all 151 patients. The sensitivity, specificity, positive predictive value (PPV) and negative predictive value (NPV) of the 16S rDNA PCR were calculated using the clinical diagnosis of IE as the reference standard. The values obtained are as follows: sensitivity = 67 %; specificity = 91 %; PPV = 96 % and NPV = 46 %.

**Table 3:** 16S rDNA PCR results in patients with and without clinical diagnosis of IE

	Patients with IE	Patients with no IE	Total
16S rDNA PCR +	78	3	81
16S rDNA PCR −	38	32	70
Total	116	35	151

### Additional value of broad-range 16S rDNA PCR in culture-negative IE

**Fig. 3:**
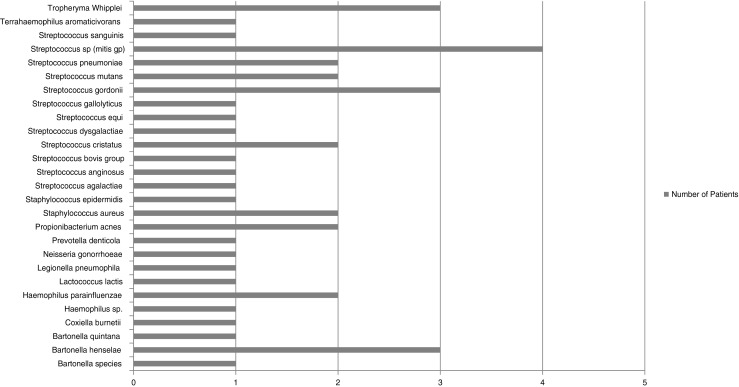
Organisms identified to the species level by broad-range 16S rDNA PCR and sequencing of valve tissue from 43 culture-negative IE patients

Sixty-nine patients had a final clinical diagnosis of IE but no significant organism was grown from blood or valve tissue (culture-negative IE). Forty-three of these patients were positive by 16S rDNA PCR on valve tissue; therefore, the PCR assay has provided additional diagnostic value in 62 % of culture-negative IE cases. The organisms identified in these patients are shown in Fig. 3. The remaining 26 culture-negative IE patients were also PCR-negative; however, two of these had organisms seen on Gram stain or by histopathological stains, and one patient had positive serology for *Bartonella quintana*.

## Discussion

This service evaluation has demonstrated that 16S rDNA PCR detects an organism in the majority (75 %) of cases of culture-proven IE. The most frequently detected organism was *Streptococcus* spp., particularly the *Streptococcus mitis* group. Further identification of the species within this group is problematic due to the high level of similarity in 16S rDNA sequences. Twelve culture-positive IE cases were PCR-negative, and coagulase-negative staphylococci (CNS) were the most frequently detected organism in this group. PCR may have failed to detect the causative organism in these cases because resection was performed some time after the blood cultures were taken. False-positive culture results are also a possibility. In six cases that were positive by culture, the PCR identification was discordant (Table 2). Five of these patients would appear to have discrepant results because of inaccurate identification of the organism by phenotypic methods. However, patient 76 would appear to have truly discordant results, but the *Streptococcus* spp. identified by PCR appears to be the most likely cause of IE, and the organisms grown on culture are more likely to be contaminants.

The sensitivity, specificity, PPV and NPV of the 16S rDNA PCR were calculated using clinical IE as the reference standard. The sensitivity (67 %) and NPV (46 %) demonstrate that PCR does not detect an organism in all clinically diagnosed cases of IE; this could be due to treated IE, sample taken late in infection, fungal IE or bacterial species that are not detected by this 16S rDNA PCR. However, the high specificity (91 %) and PPV (96 %) are likely to be biased because the clinical diagnosis of IE in some cases may have been partially based on the positive PCR result.

The 16S rDNA PCR assay has provided additional diagnostic value in 43/69 (62 %) of culture-negative IE cases. In addition to this, we have provided data on the causative organism in culture-negative IE cases from this series of UK and Ireland patients. These data were not previously available from any other published studies. The most frequently detected organism in culture-negative IE was *Streptococcus* spp., but no single species predominated. A wide range of bacterial species were detected by 16S rDNA PCR, including a number of unusual and fastidious organisms, such as *Bartonella* spp., *Coxiella* spp. and HACEK organisms, which is in agreement with recent data from other countries [2, 12, 13]. However, what is particularly striking is that three patients had IE caused by *Tropheryma whipplei*; this is consistent with other European studies, which have also reported relatively high numbers of patients with this rare organism [2, 14–16]. Such cases are unlikely to meet major criteria for IE and will only be diagnosed by PCR, because, unlike *Bartonella* spp. and *Coxiella* spp., serological tests are not available. A previous study has already demonstrated that specific PCR assays for *Tropheryma whipplei*, *Bartonella* spp. and *Coxiella burnetii* are more sensitive than broad-range 16S rDNA for diagnosing culture-negative IE from blood samples [2] and are particularly important for diagnosing culture and serology-negative IE when valvular material is not available. The data from our series indicate that a specific PCR assay for *Streptococcus* spp. would also be valuable.

In conclusion, all explanted heart valves sent for routine clinical microbiology investigations should be examined by PCR when cultures are negative, as this increases the diagnostic yield. The impact of positive 16S rDNA PCR results in cases of culture-negative IE and the potential cost savings associated with this (e.g. by rationalising antibiotics) have not been addressed, as they are outside the scope of this service evaluation. However, this type of analysis is important for understanding the real benefit of what is perceived to be an expensive laboratory investigation.
